# Replacement of clarithromycin with azithromycin in triple therapy regimens for the eradication of helicobacter pylori: A randomized clinical trial

**Published:** 2014-06-25

**Authors:** A Khoshnood, P Hakimi, H Salman-Roghani, M Reza Mirjalili

**Affiliations:** Department of Internal Medicine, Faculty of Medicine, Shahid Sadoughi University of Medical Sciences, Yazd, Iran

**Keywords:** Helicobacter Pylori, Eradication, Azithromycin, Clarithromycin

## Abstract

Abstract

Background: Eradication of helicobacter pylori is important for treatment of GU but an ideal regimen is not available. HP is resistant to metronidazole and clarithromycin. Clarithromycin is expensive and is not available in under developing countries. This study aimed to compare two regimens containing clarithromycin or azithromycin.

Methods: Totally, seventy-eight patients with GU (confirmed with endoscopy) and infection of HP (Confirmed by Rapid Urease Test (RUT)) were allocated to one of the groups of study (35 participants in each group). Two weeks regimen of Clarithromycin (2×500 mg) + Amoxicillin (2×1 gr) + omeprazole (2×20 mg) was administered for group A of patients while group B got a 10 days regimen of Azithromycin (1×250 mg) + 14 days Amoxicillin (2×1 gr) + omeprazole (2×20 mg). At the end of the treatment course, the patients were evaluated according to the side effects of the drugs. In addition, two months after the end of therapy, patients underwent endoscopy and biopsy to evaluate HP eradication.

Results: After two weeks, the side effects of the drug were: Nausea 8 patients in group A and 7 patients in group B, Diarrhea 2 patients in group A, 3 patients in group B and vomiting 2 patients in group A, 3 patients in group B. There were no serious side effects in any group. Eradication rate in group A was 82.9% (based on per protocol analysis (PPA)) and 84.6 % (intention to treat (ITT)). In group B, eradication rate was 77.1 % (PPA) and 79.5 % (ITT) (P=0.55).

Conclusion: Based on our study results, azithromycin can be used in HP eradication regimen because of its similar efficacy to clarithromycin but also have lower cost, side effects and resistance.

## Introduction

Helicobacter pylori infection is one of the most prevalent gastro intestinal infections worldwide but its prevalence in different countries and population is different, it also has a direct relationship with low socioeconomic situation [**1-4**]. In developed societies, the prevalence is about 20%-50% while in developing countries about 80% of adults are infected [**5**]. In Iran reports show that 70.1% of people with gastritis, 86.2% of duodenal ulcer patients and 70% of gastric ulcer patients are infected with helicobacter pylori that will increase by age [**6,7**]. Although helicobacter pylori infection is asymptomatic in about 90% of the patients but symptomatic ones are related to diseases like gastritis, peptic ulcer (duodenal or gastric), gastric carcinoma and gastric lymphoma [**8**]. Infection for more than ten years increases the risk of extra cardia gastric carcinomas three times more. Then its eradication is important for lowering related morbidity [**9,10**]. 

 From the suggestion of helicobacter pylori (HP) as a GI infection about 20 years ago, diagnosis and treatment of peptic ulcer has been changing fundamentally and has become an infectious disease whose eradication with antibiotics can cause remission of the lesions [**11-13**]. 

 The current treatments of peptic ulcer have two main effects, the eradication of infection and the decrease of acid production [**14,15**]. This causes a decrease in peptic ulcer recurrence and then a decrease in complications like bleeding, perforation and malignancy development [**16,17**]. For the eradication of helicobacter pylori, there are different regimens with different drugs and duration of treatments [**18,19**]. 

 The ideal regimen should be cost effective, clinically effective, tolerable and have a short duration [**20**]. 

 Considering the increasing rate of tolerance to clarithromycin and metronidazole and low resistance to azithromycin, we designed this study to compare two different regimens of helicobacter pylori eradication with clarithromycin or azithromycin. 

 Peptic ulcer disease includes gastric and duodenal ulcers. DU and GU have several similarities in signs, symptoms and diagnosis [**21**] but also have some discrimination factors. Duodenal ulcers are commonly at the first three centimeters of pylorus and are mostly benign, while gastric ulcers can indicate the cancer formation [**22**]. 

 Acute helicobacter infection can cause temporary hypochlorydia, but chronic infection can stimulate gastrin and inhibition of somatostatine and then stimulation of acid secretion and increase in basal acid output (BAO) [23,24]. The bacteria would not invade the mucosa but adhere to epithelial cells and cause mentioned inflammatory response [**24,21**]. 

 Epidermal Growth Factor (EGF) and Transforming Growth Factor Bata (TGFβ) are potent inhibitors of acid secretion and protectors of mucosa growth, HP eradication can cause EGF secretion and ulcer recovery [**21**]. Evidence shows that only species of HP that produce cytotoxins related to Cag A gene can be pathogen. About 85%-100% of the patients with Duodenal Ulcer (DU) have this type of HP infection [**25**]. 

 Helicobacter Pylori is a gram-negative bacillus that lives in the gastric mucosa, spiral shape and also flagellums of this bacterium causing motility in the gastric mucosa. Production of urease causes its resistance to acids. Human is the only reservoir of this bacterium [**26**]. 

 Azithromycin is an antibiotic from the new macrolides class that inhibits protein synthesis in bacterium and is based on drug concentration; it can be bacteriostatic or bactericide. This drug has favorable gastrointestinal absorption as it works against both gram negative and positive bacteria with good distribution in all body organs except for CNS, will not be metabolized and its clearance is mainly done by the bile [**27**]. 

 Diagnosis of HP is by non-invasive or invasive (endoscopy and biopsy) ways. Non-invasive tests include UBT (Urease Breath Test), serologic tests and stool antigen detection test. Invasive tests are Rapid Urease Test (RUT), biopsy and culture that specimen will get by endoscopy and biopsy from antrum. The best test is RUT with 80%-95% sensitivity and 95%-100% specificity [**28**]. 

 Histological study of specimen obtained by biopsy of antrum is the gold standard of diagnosis with sensitivity and specificity of more than 95% and 98% respectively that can show inflammation, metaplasia, dysplasia or even neoplasia [**28,29**]]. 

 The treatment of peptic ulcer starts with HP eradication that must be accompanied by PPI (Proton Pump Inhibitors) like Omeprazole. In addition, the patient must avoid smoking and alcohol use. Eradication of HP is difficult and multiple antibiotics must be used for weeks [**30**]. 

 At present, there are several treatment protocols for treatment. Standard protocols are Omeprazole+metronodazole+amoxycilin+tetracycline and clarithromycin+amoxycilin+omeprazole. Latest guidelines in US and Europe suggest two weeks of antibiotic treatment [**31-33**]. 

 Confirmation of the eradication is done by non-invasive tests with high sensitivity and specificity. Urea Breath Test (UBT) is the choice. Stool antigen test is another alternative test; eradication must be confirmed at least six weeks after the end of treatment. 

 Resistance of HP to clarithromycin has been increased to 15%-20% based on some recent studies and some studies recommended that in regions whose resistance to clarithromycin is higher than 20% or the patient got a macrolide previously, triple therapy with clarithromycin must not be used. 

 Considering the lack of evidence on the rate of resistance to clarithromycin in our community and also the lack of evidence which compares the effectiveness of azithromycin with clarithromycin we designed this study to compare the effectiveness of regimens with clarithromycin or azithromycin on helicobacter pylori eradication.

## Materials and Methods

This randomized clinical trial was carried out from October 2010 to September 2011 in Shahid Sadoughi Hospital and Khatam-al-Anbia Clinic, Yazd, Iran. After the confirmation of the university’s institutional ethics committee, the study was registered in clinical trials.gov as NCT01667692. Thirty-five patients were needed for each group (with consideration of the following parameters: p<0.05 as significance, test power of 80%, d=1.5 and based on previous studies S=2). Patients were selected from people who presented in the Gastroenterology Clinic of Shahid Sadoughi Hospital and Khatam-al-Anbia Paraclinic of Shahid Sadoughi University of Medical Science, have proved duodenal or gastric ulcer by endoscopy and biopsy and had infection of HP by RUT. A written consent was taken from all the patients before starting the study. The indication of treatment was based on the Health Institute of Europe guideline including peptic ulcer, chronic gastritis, maltoma and intestinal metaplasia. Before the study, block randomization was performed according to age and sex of the patients on 78 who did not have study exclusion criteria and did not prove any HP infection. 

 Exclusion criteria of study were age below 18 and more than 80, history of renal and liver diseases, sensitivity to study drugs, history of GI bleeding, history of constant NSAID using, pregnancy, use of antibiotics or bismuth for four weeks before the study. 

 Group A got a two weeks regimen of clarithromycin 500 mg at every 12 hours (Bd), Amoxicillin 1 geram at every 12 hours and omeprazole 20 mg at every 12 hours. Group B got a 10 days regimen of Azithromycin 250 mg daily, two weeks of amoxicillin 1 geram at every 12 hours and omeprazole 20 mg at every 12 hours. Drugs were obtained from the same brand. 

 During the study, patients were followed by phone calls so as not to forget to take their drugs. After finishing the treatment period, the patients were invited to the clinic for checking drugs’ side effects. Patients were requested to retain their drugs sachets and bring them to the clinic. While counting remnant drug, the patients` compliance was estimated. Patients who have used more than 80% of their administered drugs were taken as good compliance. Side effects like headache, nausea, vomiting, abdominal pain and dizziness was detected by self-report of patients. In addition, the clinical examination according to rash and epigastric pain was done. Two months after the treatment completion, all endoscopy and biopsy was done for all the patients. One sample from antrum and another from body of stomach were obtained. All specimen stained by modified Gimsa staining and studied by a pathologist was blinded to the patients’ group.

The patients’ demographic information, smoking, drug history and finding of clinical examinations, endoscopy, RUT and results of biopsy two months after the treatment completion were registered in a questionnaire. 

 All the registered data were transformed into SPSS 15 software and analyzed by Chi-square, Fisser exact test and T-test. Significant level was taken as P<0.05.

## Results

**Fig. 1:**
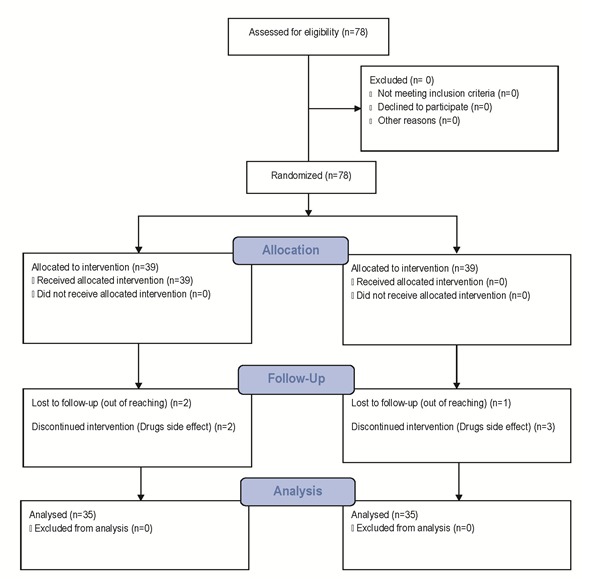
Flow of the study

Totally, 78 patients were included in the study (39 patients from each group). Two patients in group A and one patient in group B discontinued treatment because of drugs side effects. Moreover, two patients from group A and three patients from group B did not attend the follow up session (lost to follow up). Finally, 70 patients (35 from each group) were included in the analysis. The flow of the study is indicated in the Fig 1.

 The mean age of group A was 41.8±14 and in group B was 41.26±14.39 (P=0.87; T-test). Moreover, the difference between the groups according to sex was not significant (0.329). In addition, groups according to smoking was not significant (P=1.00). 

 The prevalence of drug side effects was assessed by two statistical methods of PPA (Per Protocol Analysis) and ITT (Intention To Treat). In the PPA method, 25.7% of group A patients and 31.4% of patients in group B had Side effects (P=0.59). In ITT method, these values were 23% and 28.2% in groups A and B respectively (P=0.61). 

 The rate of HP eradication also was assessed by ITT and PPA analysis methods that were not significantly different between groups (P=0.55 and P= 0.55 respectively) (**Table 1**). 

**Table 1:** Drugs side effects in groups of study based on Pre Protocol Analysis (PPA) and intention to treat (ITT) and related P-Values

Groups/Side Effect	A 35	B 35	P-Value (Chi-Square)
Per Protocol	9	11	0.59
Analysis (PPA)	25.5%	31.4%	
Intention To Treat	23%	28.2%	0.61

## Discussion

Helicobacter pylori infection is an important risk factor of peptic ulcer, chronic gastritis, maltoma and gastric adenocarcinoma and its eradication can be effective in the treatment and prevention of these diseases [34,35]. Several treatment regimens were suggested but none of them was ideal. Also, several studies were done on the type of drugs, dosage and duration of treatment that had contradictory results [**36**]. 

 Several factors were considered in the treatment regimens; cost of drugs, effectiveness, side effects and duration of drug use is needed [**36**]. Rate pf HP eradication has been estimated to more than 95% until 1995 [**37**] by using the triple regimen of PPI, amoxicillin and clarithromycin, but this regimen now has a success rate lower than 80%, that the most common cause of failure is drug resistance [**38**]. Because of this fact, searching for a newer generation of drugs, especially a new generation of macrolides like azithromycin, has been considered by researchers [**39**]. Azithromycin is resistant in acidotic environment of stoma, has a longer half life and a higher concentration in stomach tissues after oral administration of 500 mg. Its concentration will be stable on 90 mg in stomach tissue that is higher than its concentration in the plasma [**40,7**]. 

 In our study based on PPA analysis HP eradication regimen with azithromycin and clarithromycin had 79.5% and 83.9% success rates, respectively (P=0.55) and in ITT analysis had 79.5% and 84.6% success rates respectively (P=0.555). Also, there was no significant difference between two groups according to drugs side effects. 

**Table 2:** HP eradication Success rate in groups of study based on Pre Protocol Analysis (PPA) and Intention To Treat (ITT) and related P-Values

Groups/Eradication Success	A 39	B 39	P-Value (Chi-Square)
Per Protocol	29	27	0.55
Analysis (PPA)	82.9%	77.1%	
Intention To Treat	84.6%	79.5%	0.55

 In 2002, Solivan et al results showed the results in their study based on the fact that clarithromycin had better responses contrasting with azithromycin and then azithromycin was not suggested in that study [**42**]. 

 In 2003, Laurent et al mentioned that the success rate of clarithromycin was calculated to 72% while azithromycin only had 38% success rate [**34**]. In 2008, Fernando et al reached similar results, while HP eradication rate of azithromycin was 38% and, based on this study results, azithromycin was not suggested except for its low cost and good compliance [**43**]. The results of these studies are not concomitant with our study. 

 In their study, Avec et al calculated the eradication success rate of regimen with azithromycin at about 78% and clarithromycin at 81%, which were similar to our study results [**44**]. In Russia, after the comparison of eradication regimens containing azithromycin with regimen that included metronidazole, Ivashkin et al concluded that azithromycin could be an effective drug in HP eradication treatment regimens [**39**]. In a Meta analysis done by Dong et al in China, in 2009, after the inclusion of fourteen studies in the Meta analysis, it was concluded that azithromycin is an effective drug in HP eradication with low side effects [**45**]. Results of these studies are concomitant with our study. 

 Regarding cost effectiveness, in Iran, the cost of drugs that was used in our group A (regimen with azithromycin) will be of about $ 1.5 while this cost for drug regimen of group B is of about $ 4.5. 

## Conclusion

It seems that azithromycin can be used in HP eradication regimen that has similar efficacy to clarithromycin but also has lower cost and side effects. Using this drug will not increase the success rate of HP eradication but also its lower side effects and the cost effectiveness will not justify its using. Then, based on our study, the suggestion will be made to include this drug in the treatment drug regimens of helicobacter pylori. Also, we suggested the combination of other drugs like metronidazole with azithromycin to evaluate the success rate of eradication. 
